# Multiple Mechanisms Involving in Radioresistance of Nasopharyngeal Carcinoma

**DOI:** 10.7150/jca.39354

**Published:** 2020-04-25

**Authors:** Yuting Zhan, Songqing Fan

**Affiliations:** Department of Pathology, The Second Xiangya Hospital, Central South University, Changsha, Hunan, China.

**Keywords:** nasopharyngeal carcinoma, radioresistance, mechanism

## Abstract

Nasopharyngeal carcinoma (NPC) is the malignant tumor with ethnic and geographical distribution preference. Although intensity-modulated radiotherapy (IMRT)-based radiotherapy combined with chemotherapy and targeted therapy has dramatically improved the overall survival of NPC patients, there are still some patients suffering from recurrent tumors and the prognosis is poor. Multiple mechanisms may be responsible for radioresistance of NPC, such as cancer stem cells (CSCs) existence, gene mutation or aberrant expression of genes, epigenetic modification of genes, abnormal activation of certain signaling pathways, alteration of tumor microenvironment, stress granules (SGs) formation, *etc*. We conduct a comprehensive review of the published literatures focusing on the causes of radioresistance, retrospect the regulation mechanisms following radiation, and discuss future directions of overcoming the resistance to radiation.

## Introduction

Nasopharyngeal carcinoma (NPC) is the malignant tumor with a unique pattern of geographical distribution, mainly in Southern China and Southeast Asian and North African countries 1. It was estimated by Global Cancer Statistics 2018 that there would be about 129,079 and 72,987 new cases and cancer-related deaths of NPC, accounting for 0.7% and 0.8% of all new cancer patients and dead ones respectively 2. Based on World Health Organization (WHO) histological classification, NPC is classified into keratinizing squamous cell carcinoma (type I) and nonkeratinizing carcinoma, the latter of which is subdivided into differentiated (type II) and undifferentiated (type III) kinds. Almost all patients with non-keratinized NPC in Southern China are infected with Epstein-Barr virus (EBV) 3, 4. Overall prognosis has dramatically improved over the past decades with the development of imaging (for more accurate disease staging), radiotherapy, chemotherapy and targeted therapy. Among multiple treatments, intensity-modulated radiotherapy (IMRT) is the current standard of treatment for NPC achieving good tumor coverage and healthy tissue sparing, and thus permits dose escalation as well as decent local control for most patients 5-7. The regional control rate for IMRT is nearly more than 90%, with relatively satisfying progression-free survival and overall survival for newly diagnosed patients with early stages 5, 6, 8, 9. However, radioresistance induces local failure, resulting in residual or recurrent tumors for some patients, and is the leading cause of NPC treatment failure 10, 11. For locally advanced recurrent nasopharyngeal carcinoma patients, the local control rate, progression-free survival, and 3-year overall survival are really too significant to be ignored, with only 44.3%, 17.5% and 47.2% respectively 12. Resistance to radiotherapy can occur for a variety of reasons, such as gene mutation or aberrant expression of genes, epigenetic modification of genes, abnormal activation of certain signaling pathways, alteration of tumor microenvironment, stress granules formation, *etc*.

A better understanding of the mechanisms contributing to radioresistance in NPC will enable the development of therapeutics that promote tumor remission and reduce the radiation dose necessary, achieving tumor control, thereby improving the patients' survival rate and life quality. We conduct a comprehensive review of the published literatures focusing on the initial and influential factors of radioresistance, and discuss future directions of overcoming this bottleneck.

## The roles of cancer stem cell (CSC) in radiotherapy resistance of NPC

Ionizing radiation can direct target nuclear DNA, which can either cause DNA damage by direct DNA ionization or indirectly induce DNA injury by stimulating reactive oxygen species (ROS) production. Various consequences for cancer cells can be induced by DNA damage such as apoptosis, mitotic catastrophe, autophagy, necrosis and cell senescence 13-15. Nevertheless, radioresistance inevitably occur for some patients, and cancer stem cells (CSCs) and the metabolic reprogramming of cancer cells are supposed to partially responsible for that. The main theory of CSCs is that tumor growth is fueled by a small amount of cells conferring to the capacity to self-renew and give rise to highly proliferative cells forming the bulk of the tumor 16-18.

CSCs are identified in various kinds of malignant tumors including brain tumor 19, 20, breast cancer 21, colon cancer 22, melanoma 23, *etc*. Over the past years, increasing number of studies has been carried out to isolate and identify CSCs from human NPC cells and tissues 24. Label-retaining cells (LRCs) are considered to be putative stem cells characterized by slow-cycling and dividing infrequently. These stem-like LRCs that exist in both nasopharyngeal epithelia and NPC tissues can be recruited into the cell cycle to participate in xenograft tumors of nude mice 25, 26. A small group of NPC cells (side population cells, SP cells) can be isolated and identified by flow cytometry analysis with stem cell characteristics, such as proliferation, self-renewal, and differentiation, which have a strong ability to form tumors 27. Some cell labels may serve as potential molecular markers for further characterization of CSC including cytokine 19, CD44v6, ALDH1A1, CD44, CD24, CD133, OCT4, SOX2, NANOG 24, 27-33.

Compared to the mass of tumor cells, CSCs confer to higher radioresistance and possess specific molecular properties protecting them from radiation-induced damage. As is known, it is crucial and significant for cell death or repair of the efficacy of the DNA repair machinery activated by the DNA damage signaling network. A high DNA repair capacity has been described for CSCs in different tumor entities and mainly attributed to the activation of the ATR-Chk1 and ATM-Chk2, moreover, the mechanisms of CSC radioresistance also includes protection from oxidative stress by ROS scavenging, activation of anti-apoptotic pathways, protection by microenvironmental niche 34. Recent studies have demonstrated that the phenotype of CSCs may be quite associated with the epithelial-to-mesenchymal transition (EMT), and cancer cells that undergo EMT have been shown to acquire stemness and undergo metabolic changes 35, 36. EMT can endow cancer cells with features such as migration, invasion, resistance to anoikis, chemoresistance and radioresistance, enabling the initiation of metastasis 13, 37. Ionizing radiation can induce EMT process; in return, EMT process can promote radioresistance. EMT qualifies mesenchymal properties to epithelial cells characterised by losing epithelial markers (such as E-cadherin, α-cadherin) and acquiring mesenchymal markers (such as Vimentin, fibronectin, N-cadherin) 38-41. EMT process associates closely to the phenotype of CSCs, and can promote radioresistance. Various molecules and signaling pathways involve in the regulation of EMT, CSCs and radioresistance, and we will discuss it in the following parts.

## Multiple signaling pathways are involved in radiotherapy resistance of NPC

**Figure 1:**
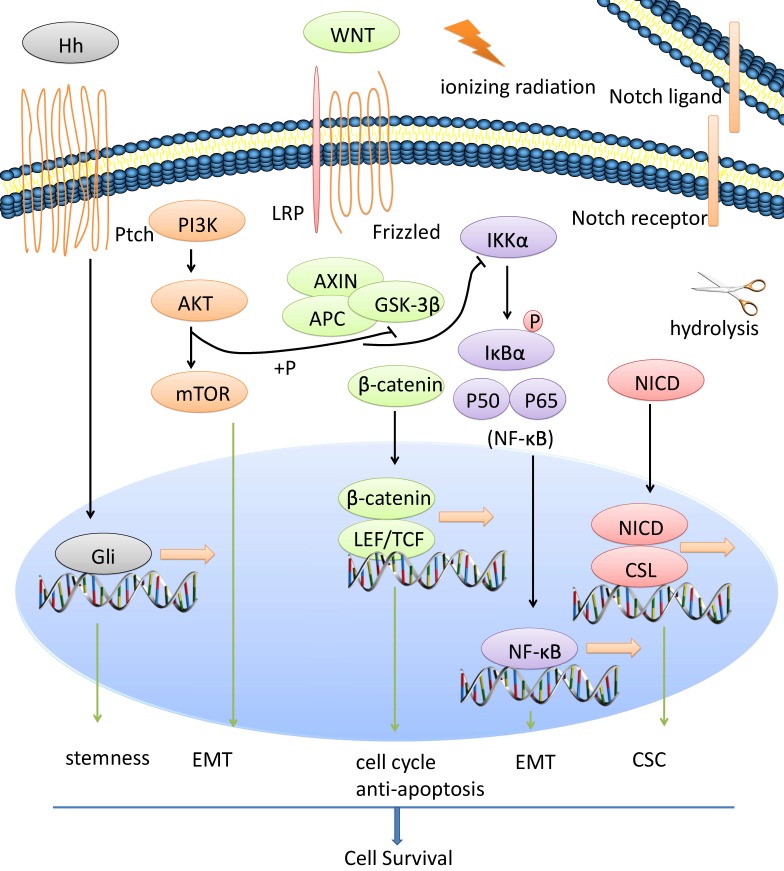
** Multiple signaling pathways are involved in radiotherapy resistance of NPC.** CSCs, EMT process and radiotherapy resistance of NPC is regulated by a network of signaling pathways such as Wnt/β-catenin, NF-κB, Notch, AKT, Hedgehog, *etc*.

CSCs, EMT process and radiotherapy resistance of NPC is regulated by a network of signaling pathways such as Wnt/β-catenin, NF-κB, Notch, AKT, Hedgehog, *etc*. We show the general view in Figure 1.

### Wnt/β-catenin signaling pathway and radioresistance of NPC

Wnt/β-catenin signaling pathway maintains cell stemness and induces radioresistance in several human cancers including NPC 42-45. Wnt signaling is detailedly discussed in many reviews 46. Briefly, the canonical pathway is activated upon binding secreted Wnt to its receptors Frizzled (Fzd) and low-density lipoprotein receptor-related 5/6 (LRP5/6), following destruction complex (including Axin, CK1α, GSK3β and APC) destroyed and stabilization of β-catenin. Accumulated β-catenin can enter the nucleus, displace Groucho and bind to LEF/TCF (lymphoid enhancer-binding factor/T-cell factor) and eventually promote multiple target gene transcription. One of the Wnt protein family members, Wnt2B, may have potential function in NPC radioresistance. Wnt2B expression is upregulated in CNE-2 cells (NPC cells) following ionizing radiation treatment, and is significantly increased in radioresistant CNE-2 cells. Silencing of Wnt2B reduces the invasion and metastasis ability of 5-8F cells (NPC cells) and can significantly inhibit the radioresistance of 5-8F cells, following downstream β-catenin decreased and p-GSK-3β increased. Moreover, clinical statistics show that up-regulation of Wnt2B expression is correlated with an advanced clinical stages of NPC patients, and for the same patient, increased Wnt2B is observed after radiotherapy 47-49. Compared with radiosensitive NPC patients, higher expression of β-catenin is revealed in radioresistant NPC by immunohistochemical staining. Also, β-catenin overexpression can decrease the radiosensitivity of CNE-2 cells with more colony-formation and higher surviving fraction values. Following radiation, β-catenin overexpression decreases GSK-3β expression and increases Cyclin D1 expression, promotes viability of CNE-2 cells post-radiation. Radiation may also increase the TCF/LEF transcriptional activity of CNE-2 Cells overexpressing β-catenin 45. Moreover, other signaling pathways may have crosstalks with β-catenin to promote radioresistance of NPC 50.

### NF-κB signaling pathway and radioresistance of NPC

Abnormal NF-κB signaling and genetic mutations in NF-κB signaling‐associated factors impact the tumorigenicity, proliferation, chemoresistance and radioresistance of multiple kinds of cancer including NPC. Normally, a varies of stimuli can initiate NF-κB signaling such as cytokines, growth factors, reactive oxygen species and ionizing radiation. Upon the stimulations, the inhibitor of κB kinase (IKK) complex phosphorylates IκBα, leading to its ubiquitination and proteasomal degradation. Thus, the free NF-κB dimers can accumulate and translocate from the cytoplasm to the nucleus, bind to DNA and promote transcription of its target genes^51^, ^52^. Activated IKKβ can promote Twist1 expression, contributing to the EMT phenotype, and TNFα stimulates p65 to bind to the human Twist1 promoter and regulate its transcription 53. NF-κB p65 knockdown can reverse the EMT process in nasopharyngeal carcinoma CNE-2 stem-like cells. When NF-κB p65 knockdown, the levels of the EMT markers N-cadherin and Vimentin are decreased, whereas E-cadherin level is increased 38. Activation of NF-κB confers tumor resistance to radiotherapy, and many molecules may interact with NF-κB signaling to regulate radiosensitivity of NPC 54-56.

### Notch signaling pathway and radioresistance of NPC

In brief, Notch signaling is activated when a ligand (including DLL1, DLL3, DLL4, JAG1, and JAG2) binds to a Notch receptor (including Notches 1~4), which undergoes proteolytic cleavage by ADAM family proteases and γ-secretase, releasing the Notch Intracellular Domain (NICD). The NICD can translocate to the nucleus where it binds to the ubiquitous transcription factor CBF-1/suppressor of hairless/Lag1 (CSL) and converts the complex from a repressor to an activator of Notch target genes 57. The Notch signaling pathway is involved in CSC and radiosensitivity of NPC. DAPT, a kind of Notch inhibitor, can inhibit NPC cell proliferation *in vitro* and *in vivo*, deplete SP cells in NPC cell lines, reduce colony formation and induce apoptosis. Targeting Notch signaling seems to have a decent clinical prospect 58, 59.

### AKT along with its crosstalk and radioresistance of NPC

AKT has been shown to play a crucial role in oncogenesis, migration, invasion, chemoresistance and radioresistance of various malignant tumors including NPC 60. Epithelial cell adhesion molecule (EpCAM) can promote NPC cell EMT process with upregulation of N-cadherin, Vimentin and β-catenin along with downregulation of α-catenin and E-cadherin. EpCAM can also regulate the stem-like phenotype accompanying enhanced levels of CD44, OCT4, Nanog and ABCG2. Treatment with the AKT inhibitor MK2206 or mTOR inhibitor rapamycin can almost completely abrogate the EpCAM-induced invasiveness and stemness 61. In the cohort of NPC patients, the levels of phospho-AKT are significantly higher in the radioresistant NPCs than those in the radiosensitive NPCs 62, 63. Importantly, AKT is the common point of convergence of multiple signaling axis such as IL8/AKT 62, ERK/AKT 63, PI3K/ AKT/Bcl-2 64, FAK/Akt/JNK 65, PTEN/Akt/mTOR 61, 66, 67, *etc*. Many genes and non-coding RNAs can associate with AKT along with its crosstalk to regulate radioresistance of NPC.

### Hedgehog signaling pathway and radioresistance of NPC

It is significantly different in the expression of Hedgehog signaling components (PTCH1, GLI1, GLI3, and SUFU) and its targets (FOXM1, BCL2, CFLAR and SOX2) between NPCs and normal nasopharyngeal mucosal specimens. Aberrant Hedgehog pathway activation stimulates stemness marker gene (CD133, CD44, SOX2, EPCAM, LRIG1 and BMI1) expression in EBV-infected epithelial cells compared with EBV-negative carcinoma-derived cell lines. Moreover, EBV-infected epithelial cells and authentic EBV-positive carcinoma cell lines require constitutive Hedgehog pathway activity for tumor sphere formation 68, 69.

## Multiple genes are involved in radiotherapy resistance of NPC

The activation of oncogenes or the loss of tumor suppressors is playing important roles in tumor initiation and progression, which can also regulate the EMT process, CSC formation and radiosensitivity of NPCs. Recently, comprehensive studies of genes/proteins are based on three aspects: (1) Multiple genes can interact with signaling pathways to regulate radiosensitivity; (2) Various proteins are regarded as decent biomarkers for predicting the reaction for radiotherapy 70; (3) Gene fusion can induce radioresistance.

### Multiple genes can interact with signaling pathways to regulate radiosensitivity and their encoded proteins can act as useful potential biomarker

We take leukemia inhibitory factor (LIF) as an example. LIF is playing a vital role in embryonic stem cells self-renewal, reproduction, bone remodeling, the hypothalamo-pituitary-adrenal axis, the neuromuscular system, the hemopoietic system and cancer 71. On the one hand, LIF activates mTORC1/p70S6K signaling via LIF receptor, leading to increased tumor growth; LIF-mediated radioresistance may result from the inhibition of DNA damage responses (DDR) signaling; EBV-encoded LMP1 can induce LIF expression through NF-κB activation in NPC cells. On the other hand, compared with NPC patients with complete tumor remission, LIF is higher in serum samples from NPC patients who developed local recurrence after treatment. Notably, higher LIF levels were markedly correlated with poorer local recurrence-free survival 10, 72.

Many other molecules can play roles similar to LIF. They can associate with various signaling pathways, directly or indirectly binding to the signaling members, changing the phosphorylation state and affecting target gene expression. At the same time, their encoded proteins can act as potential biomarkers for predicting radiosensitivity. We summarize genes along with their associated pathways and encoded proteins in Table 1.

### Gene fusion can induce radioresistance of NPC

Genomic instability and mutation is one of the emerging hallmarks of cancer. Certain mutant genotypes confer selective advantage on a subgroup of cells, enabling their outgrowth and eventual dominance in a local tissue environment 84. Not only chromosome translocations and relevant gene fusions may contribute to tumor progression, but also the fusion protein produced by a fusion gene can be oncogenic. FGFR3-TACC3 fusion gene can trigger activation of the ERK and AKT signaling pathways, and promote cell proliferation, colony formation, and transforming ability 85. UBR5-ZNF423 fusion gene can promote NPC cell proliferation and colony‐forming ability; the growth of NPC cells with UBR5-ZNF423 rearrangement depends on expression of this fusion protein 86.

Fusion gene can also contribute to tumorigenesis, CSC-like properties, and therapeutic resistance. RARS-MAD1L1 has oncogenic potential in NPC, enhancing cell transformation abilities and promoting colony formation. Overexpression of RARS-MAD1L1 can upregulate the levels of stem cell markers (ABCG2, c-Myc, Sox2 and Bmi-1) in NPC cells and induce SP cells. RARS-MAD1L1 fusion confers NPC cells more radioresistant phenotype and they can form more colonies under irradiation 87.

## Epigenetics can regulate radioresistance of NPC

### MicroRNAs and radioresistance of NPC

MicroRNAs (miRNAs), always is defined as single stranded non-protein-coding RNA molecules with 18-25 nucleotides in length, can bind to the 3' untranslated regions (3'UTRs) of target mRNAs (in few cases bind to 5'UTR) and thus lead to mRNA degradation or translational repression. MiRNAs may not only act as either oncogenes or tumor suppressors, regulating cell proliferation, migration, invasion and metastasis, but also involve in EMT process, CSC formation and radiosensitity of NPCs, revealing decent predicting roles 88, 89.

Reviewing published literatures, the mechanisms for miRNAs regulating radiosensitivity focus on two aspects: targeting certain genes or targeting relevant pathways. MiRNAs along with their associated genes or pathways are presented in Table 2.

### Long non-coding RNAs and radioresistance of NPC

Long noncoding RNAs (lncRNAs), normally described as transcripts larger than 200 nucleotides, are not thought as transcriptional “noise” any more, and have been regarded as a new frontier in the study of human malignant cancers including NPC 99. There are basically eight mechanisms for lncRNAs to regulate biological process, extensively participating in initiation, progression and prognosis of multiple kinds of cancer 100. In the study of NPC, some lncRNAs are playing oncogenetic roles such as LINC01420 101, LOC100129148 102, NPCCAT1 103, AFAP1-AS1^104^, *etc*. Enhanced expression of them can either promote cell growth, proliferation, migration and invasion, or predict markedly poorer survival time (LOC100129148 and AFAP1-AS1), which may function as a competitive 'sponge' for miRNA (LOC100129148 and AFAP1-AS1) or directly binds to target gene mRNA 5'UTR and regulates its protein level (NPCCAT1). Whereas, some lncRNAs such as LINC0086 105 and MEG3 106, can act as tumor suppressors to regulate cell proliferation, colony formation, cell cycle and apoptosis by directly interacting with miR-214 (LINC0086) or by p53 signaling cascade (MEG3).

LncRNAs also participate in the regulation of EMT process, CSC formation and radiosensitity of NPCs. MALAT1, widely studied in various kinds of malignant tumor, is demonstrated to regulate proliferation and invasion of NPC cells; knocking down MALAT1 can inhibit EMT process with increased E-cadherin along with reduced N-cadherin and Vimentin; in-depth study reveals that the regulating roles of MALAT1 is through de-repressing Capn4 by sponging miR-124 107. So as TUG1 108 and FEZF1-AS1 109 are presented to influence EMT process in NPC. Besides, THOR expression was positively correlated with the expression of stemness markers (ALDH1 and Nanog) especially in spheroids of NPCs, whereas knockdown of THOR attenuates proliferation, migration, and self-renewal ability of NPC stemness-like cells 110. Additionally, it is reported that knocking down PVT1 enhances the radiosensitivity of NPC cell lines, promotes the apoptosis of NPC cells induced by radiotherapy via caspase and results in diminution of the DNA repair ability through the ATM-p53 pathway 111. Knocking down ANRIL can also repress proliferation, induce apoptosis, and enhance radiosensitivity in NPC cells via functioning as a miR-125a sponge 112.

### DNA methylation and radioresistance of NPC

DNA methylation, a common mechanism of epigenetic alterations, may be correlated with NPC. Promoter methylation of tumor suppressor is playing a significant role in NPC development and progression. Large sample clinical data suggest that RASSF1A (RAS association domain family protein 1A) promoter methylation is related to clinical stage, lymph node status, distant metastasis, and T classification of patients with NPC 113. Signaling pathways can be epigenetically regulated by methylation of cellular genes, for example, methylation of Wnt signaling regulators (SFRP1, 2, 4 and 5, DACT2, DKK2 and DKK3) can be detected in NPC patients 114. DNA methylation can regulate radiosensitivity as well. MiR-24, a potential radiosensitizer, is positively correlated with the sensitivity of NPC to ionizing radiation. However, miR-24 is downregulated after irradiation, for which mir-24-1 (miR-24 genes) promoter hypermethylation may account 115.

## EBV as an oncogenic virus to promote radioresistance of NPC

EBV, a kind of well-known oncogenic viruses, contributes to the aggressiveness of NPC. There is a close relationship between EMT process, CSCs along with radioresistance and EBV products including EBV latent membrane proteins (LMPs), EBV nuclear antigens (EBNAs), EBV-encoded RNAs (EBERs) and viral microRNAs 3.

LMP1 is a primary oncoprotein encoded by EBV. LMP1 consists of a short cytoplasmic N-terminustail, six transmembrane domains, and a long cytoplasmic C-terminus that contains C-terminal-activating regions (CTARs) including CTAR1, CTAR2, and CTAR3. Each CTAR can serve as docking sites for cellular adaptors to trigger a series of signaling pathways such as NF-κB, p38/MAPK, JAK, JNK and PI3K/AKT 116. Genomic instability is associated strongly with tumor formation, notably, LMP1 may repress DNA repair and contribute to genomic instability in human epithelial cells by triggering the PI3K/Akt pathway to inactivate the FOXO3a (The Forkhead box class O) and decrease DDB1 (DNA damage-binding protein 1) 117. In addition to epithelial cells, LMP1 may repress DNA double strand breaks repair in NPC cells induced by irradiation through inhibiting DNA-dependent protein kinase (DNA-PK) phosphorylation and activity. LMP1 can mediate radioresistance by suppressing the DNA damage response through DNA-PK/AMPK signaling 118. Moreover, LMP1 induces the development of CSCs and leads to radioresistance in NPC. LMP1 positively regulates the expression of the CSC marker CD44 and several stemness-related genes (Nanog, Oct4, Bmi-1 and SOX2), increases the number of SP cells, enhances the self-renewal properties and promotes the *in vivo* tumor initiation ability. Inactivation of the p53-mediated apoptosis pathway may be responsible for the radioresistance 119. LMP1 overexpression leads to significantly higher expression of miR-155, which can increase survival fraction of NPC cells after radiation and decrease cell apoptosis 120.

Viral microRNAs are also important to radioresistance for NPC. MiR-BART4 expression was elevated in EBV positive NPC tissues compared with EBV negative NPC tissues, and the expression of miR-BART4 in NPC tissues was increased with advanced clinical stage, lymph node metastasis as well as poorer differentiation. Moreover, the expression of miR-BART4 was highly expressed in sensitive NPC tissues, and down-regulation of miR-BART4 can increase the radiosensitivity of NPC cells 121. MiR-BART1 is found dramatically increased in advanced clinical stages of NPC patients. MiR-BART1 compels EMT with the decreased expression of E-cadherin and increased expressions of N-cadherin and vimentin. Mechanistically, miR-BART1 directly targets the cellular tumor suppressor PTEN and activates PTEN-dependent pathways 41.

## Tumor microenvironment are involved in radiotherapy resistance of NPC

Radiation not only induces changes in cancer cells, but also remodels the tumor microenvironment (TME). The TME comprises extracellular matrix (ECM, including cytokines, growth factors, hormones, extracellular matrix, *etc*.) and multiple cell types (including fibroblasts, macrophages, vascular endothelial cells, immune cells, etc). There is a complex crosstalk between cancer cells and tumor-resident cells, facilitating tumor initiation, progression, and metastasis. Importantly, radiotherapy can change tumor cells and TME, inducing EMT process along with CSCs formation, creating favourable TME, and thereby leading to radioresistance 13, 122.

Cancer associated fibroblasts (CAFs) are defined as spindle fibroblast-like stromal cells with expression of α-smooth muscle actin (α-SMA). The density of CAFs is significantly correlated with relapse as well as T stage and inversely associated with the prognosis of NPC patients 123. Moreover, combination with CAFs and tumor-associated macrophages (TAMs, characterized CD68 for pan-macrophage marker and CD163 for M2 macrophages) can also be used to predict prognosis of NPC patients. The expressions of CD163 and α-SMA are independent prognostic factors for the overall survival and progression-free survival; failure risk groups based on expression levels of CD163 and α-SMA are independent predictors for the survival of patients with NPC 124. In a study of esophageal squamous cell carcinoma, CAF-secreted CXCL1 (a kind of chemokine) confers tumor radioresistance by MEK/ERK signaling pathway and activation of DNA damage repair in a SOD1-ROS-axis-dependent manner 125. They also find CAF-promoted lncRNA DNM3OS confers significant radioresistance *in vitro* and *in vivo* by regulating DNA damage responses in a PDGFβ/PDGFRβ/FOXO1 signaling pathway-dependent manner 126.

On the one hand, microvessel density is significantly higher in the stroma of NPC tissues than chronic nasopharyngitis specimens 127, on the other hand, the vasculature of tumor exhibits altered structural and functional properties, resulting in areas of hypoxia and limited nutrient supply 122. Following radiation, tumor microenvironment regarding reoxygenation, ROS, and HIF-1 along with its target gene vascular endothelial growth factor (VEGF) or its associated pathways is complicated and dynamic. Hypoxia induces HIF-1, in turn increasing tumor angiogenesis. Besides, HIF-1 activity in tumors was more rapidly increased after radiation compared to the untreated group, whose HIF-1 activity gradually increases as tumors grows 128, 129. Targeting HIF-1 may improve efficacy to radiotherapy, moreover, the combination of radiation treatment and targeting angiogenesis may be a promising therapeutic method for the treatment of NPC; some clinical trials are in process, showing decent prospect 38, 129, 130, 131.

Radiation induced hypoxia or radiation itself can modulate the microenvironment on all levels, involving in various kinds of cells such as T cell, regulatory T cell, myeloid derived suppressor cell (MDSC), TAM, dendritic cell (DC) and NK cell, *etc* 132. Notably, EBV can interact and influence microenvironment, participating initiation, progression, prognosis chemoresistance and radioresistance of NPC patients 133, 134. A distinct TME is raised with the coexistence of tumor-infiltrating lymphocytes and EBV-infected NPC cells, which will facilitate tumor development along with progression and repress immune surveillance in early phase of EBV infection 135. Whereas, EBV positive NPC contains significantly more CD3, CD4 and CD8 tumor infiltrating lymphocytes compared with EBV negative NPC; EBV positive patients with coexpression of CD8 and PDL1 showed better disease free survival and overall survival 133.

## Stress granules and radioresistance of NPC

Stress granules (SGs), non-membranous structures formed in response to different stress stimuli, contain translationally-stalled mRNAs, associated preinitiation factors, and specific RNA-binding proteins 136, 137. Radiotherapy is a kind of stress stimuli for both tumor cells and adjacent normal tissues, leading to cell death via apoptosis, autophagy, mitotic catastrophe and terminal growth arrest senescence 138. SGs can confer survival advantages and chemotherapeutic or radiotherapeutic resistance to tumor cells under stress 139, 140. Radiation may damage tumor's vasculature and position tumor cells in relative hypoxic environment, inducing expression HIF-1. Benjamin J Moeller and his colleague find that 4T1 cells (mouse breast cancer cell line) can be stained for TIAR, a SG component, when exposed to hypoxia (0.5% O2) with or without reoxygenation (21% O2). Interestingly, SGs appear after hypoxic incubation and dissipate rapidly upon reoxygenation. It is speculated that tumor reoxygenation leads to enhanced translation of HIF-1-regulated transcripts secondary to SG depolymerization after radiotherapy and partially result in radioresistance 140. Besides, eIF4E has a positive role in SG formation, and the activation of eIF4E is proved in nasopharyngeal carcinoma 141, 142. Despite the lack of direct evidence that SGs assembly or disassembly participate in the radiosensitivity of NPC, many clues imply that SGs may play crucial roles in mRMA translation process and thus affect radioresistance so as to other malignant tumors 140, 143.

## Conclusions and future directions

**Figure 2:**
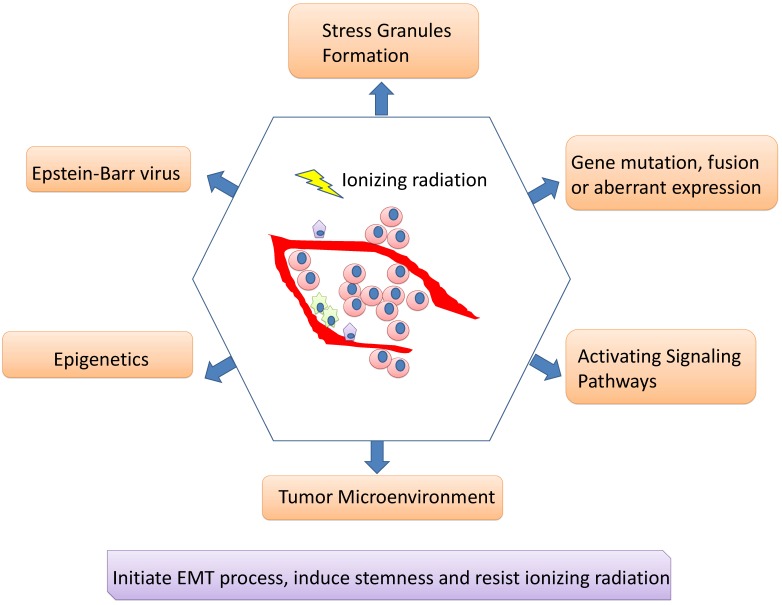
** Mechanisms involving in radioresistance of nasopharyngeal carcinoma.** A network of signaling pathways participate in the EMT process, cancer stem cell formation/phenotype as well as radiotherapy resistance. Besides, multiple genes can interact with signaling pathways to regulate radiosensitivity, and their encoding proteins can act as potential biomarkers for predicting the reaction for radiotherapy. Epigenetics can regulate radioresistance of NPC as well. Moreover, it cannot be ignored for the roles of EBV products contributing to the aggressiveness and radioresistance of NPC, along with its roles in regulating microenvironment. Stress granules formation also participates in the radioresistance of nasopharyngeal carcinoma.

Although IMRT-based radiotherapy combined with chemotherapy and targeted therapy has dramatically improved the overall prognosis of NPC patients, there are still some patients suffering from recurrent tumors and treatment failure. Radiotherapy resistance is inevitably occur for some patients and is supposed to major clinical challenge for NPC. The CSC existence or phenotype and its associated EMT process may be responsible for radioresistance; tumor cells undergoing EMT may acquire stemness and insensitive to radiation. A network of signaling pathways participate in the EMT process, CSC formation/ phenotype as well as radiotherapy resistance including Wnt/β-catenin, NF-κB, Notch, AKT, Hedgehog, *etc*. Besides, multiple genes can interact with signaling pathways to regulate radiosensitivity, and their encoding proteins can act as potential biomarkers for predicting the reaction for radiotherapy. Epigenetics can regulate radioresistance of NPC as well. A number of miRNAs and lncRNAs may not only act as either oncogenes or tumor suppressors, regulating cell proliferation, migration, invasion and metastasis, but also involve in EMT process, CSC formation/phenotype and radioresistance of NPCs, revealing predicting roles. DNA methylation, another important field of epigenetics, is also playing irreplaceable roles. Moreover, it cannot be ignored for the roles of EBV products contributing to the aggressiveness and radioresistance of NPC, along with its roles in regulating microenvironment. CAFs, hypoxia and tumor vasculature engage in microenvironment associated radioresistance as well (Figure 2).

In the future, further basic scientific research about radioresistance of NPC can be concentrated on the following aspects: (1) seeking methods to sensitize CSCs to radiation (2) blocking approaches for tumor cells to endow CSC phenotype or inhibiting EMT process (3) studying more details for a network of signaling pathways to regulate radioresistance (4) finding more biomarkers (including tissues and blood test) for predicting radioresistance and overall prognosis (5) discovering more interaction ways for genes to influence radioresistance (6) identifying new miRNAs or lncRNAs and their detailed mechanisms (7) revealing how EBV interact with cancer cells or adjacent cells to influence TME and promote NPC progression (8) illuminating how TME interact with NPC cells and induce immune escape (9) exploring how tumor cells reply to stress and association with SG assembly or disassembly (10) developing more effective sensitizer for tumor cells to radiation.

## Figures and Tables

**Table 1:** Multiple genes can interact with signaling pathways to regulate radiosensitivity and their encoded proteins can act as good potential biomarker

Genes	Relationship with radiotherapy	Associated signaling pathways	Potential biomarkers	Citation
RPA3 (Replication protein A3)	overexpression of RPA3 can enhance radioresistance and the capacity for DNA repair of NPC cells		High RPA3 expression is associated with shorter overall survival (OS) and a higher recurrence rate	11
RBM3(RNA-binding motif protein 3)	RBM3 enhance radioresistance by inhibiting the apoptotic response to radiotherapy	through the PI3K/AKT/Bcl-2 signaling pathway	RBM3 may serve as a novel factor for predicting radioresistance	64
EDA (Fibronectin extra domain A)	EDA-silenced NPC cells show enhanced radiosensitivity	by FAK/Akt/JNK signaling		70
EVI1(Ecotropic Viral Integration Site 1)	EVI1, snail, and HDAC1 can form a co-repressor complex to repress E-cadherin expression and contribute to EMT phenotype; EVI1 can regulat CSC properties	EVI1 directly bind at β-catenin promoter and activate its expression	Up-regulation of EVI1 predict unfavorable prognosis and contribute to radioresistance in NPC cells	73
ANXA1(Annexin A1)	ANXA1 downregulation may enhance the radioresistance of NPC			74
BPIFB1(Bactericidal/permeability- increasing-fold-containing family B member 1)	BPIFB1 sensitize NPC cells to ionizing radiation	BPIFB1 can inhibit VTN-mediated radioresistance		75
SHP-1 (also called PTPN-6, Protein Tyrosine Phosphatase, Non-Receptor Type 6)	Overexpression of SHP-1 lead to radioresistance with enhanced DNA DSB repair, increased S phase arrest and decreased cell apoptosis			76,77
QSOX1(quiescin sulfhydryl oxidase 1)	QSOX1 is significantly down-regulated in the radioresistant NPC cell line; QSOX1-silencing weaken the antitumor effect of radiation and enhance the radioresistance			78
SALL4 (sal‐like 4)	Inhibition of SALL4 reduce proliferation and sensitize cells to radiation	via ATM/Chk2/p53 pathway		79
HSP27 (heat shock protein 27)	HSP27 upregulation may be involved in the NPC radioresistance.			80
CKMT1(Ubiquitous mitochondrial creatine kinase 1)	NPC cells with higher CKMT1 exhibit lower radiosensitivity	through promoting phosphorylation of STAT3		81
CD166	The secreted level of CD166 with radioresistant NPC is significantly higher than that with radiosensitive NPC		the secreted protein CD166 may be can used as a biomarker for predicting the response of NPC to radiotherapy	82
PLAC8 (placenta specific 8)	knockout of PLAC8 enhance the radiosensitivity of NPC cells	by PI3K/AKT/GSK3β pathway		83

**Table 2:** MicroRNAs regulate radiosensitivity and can act as good potential biomarker for predicting radioresistance

MicroRNAs	Roles in radiotherapy	Associated signaling pathways/genes	Potential biomarkers	Citation
**MicroRNAs conferring to radioresistance**				
miRNA-324-3p	miRNA-324-3p is significantly decreased in CNE-2 cells with radioresistance compared to its parental cells	targeting WNT2B (by the 5′-UTR)		47
miR-185-3p	miR-185-3p can increase the radioresistance of NPC cells to irradiation; miR-185-3p can affect EMT process	targeting the coding region of Wnt2B; activating the WNT2B/β-catenin pathway		48
miR-19b-3p	miR-19b-3p overexpression result in decreased sensitivity to irradiation	activating the TNFAIP3/ NF-κB axis	an independent predictor for reduced patient survival.	55
miR-125b	miR-125b increment is significantly correlated with NPC radioresistance	targeting A20 and then activating the NF-κB signaling pathway	an independent predictor for poor patient survival	56
miR-205	miR-205 is significantly elevated followed the radiotherapy	targeting PTEN		67
miR‑222	miR‑222 upregulation confer radioresistance.	targeting PTEN; regulating the PI3K/AKT signaling pathway		90
miR-20a-5p	miR-20a-5p promotes NPC radio-resistance.	1)targeting Rab27B 2)targeting NPAS2; regulating Notch pathway		91,92
miR-206	miR-206 is down-regulated in radioresistant NPC cells	targeting IGF1 and inhibited the PI3K/AKT pathway		93
miR-504	miR-504 is up-regulated during different weeks of radiotherapy	targeting NRF1 and disturbing mitochondrial respiratory function	patients with high expression of miR-504 exhibits a relatively lower therapeutic effect ratio of complete response, but a higher ratio of partial response	94
**MicroRNAs contributing to radiosensitivity**				
miR-203	miR-203 is frequently downregulated in the radioresistant NPC tissues	targeting IL8 and then activating the IL8/AKT signaling pathway	its decrement significantly correlated with NPC radioresistance and poor patient survival	62
miR-23a	miR-23a is frequently downregulated in the radioresistant NPC tissues	activating IL-8/Stat3 signaling	its decrement correlated with NPC radioresistance and poor patient survival	95
miR‐24	miR‐24 suppress NPC cell viability and sensitize NPC cells to IR. miR-24 levels are significantly decreased in recurrent NPC	1)targeting Specificity protein 1 (SP1); 2)by directly regulating Jab1/CSN5 (targeting both 3'UTR and 5'UTR)	serve as prognostic markers for NPC recurrence	96,97
miR‑495	miR‑495 inhibition lead to a significant increase in the radioresistance; involved in EMT process	targeting GRP78		98

## Abbreviations

NPC: Nasopharyngeal carcinoma
IMRT: intensity-modulated radiotherapy
CSC: cancer stem cell
SG: stress granule
WHO: World Health Organization
EBV: Epstein-Barr virus
ROS: reactive oxygen species
LRC: Label-retaining cell
SP cell: side population cell
EMT: epithelial-to-mesenchymal transition
SF: surviving fraction
EpCAM: Epithelial cell adhesion molecule
DDR: DNA damage response
miRNA: MicroRNA
lncRNA: Long noncoding RNA
LMP: latent membrane protein
EBNA: EBV nuclear antigen
EBER: EBV-encoded RNA
DNA-PK: DNA-dependent protein kinase
TME: tumor microenvironment
ECM: extracellular matrix
CAF: Cancer associated fibroblast
TAM: tumor-associated macrophage
VEGF: vascular endothelial growth factor
MDSC: myeloid derived suppressor cell
DC: dendritic cell

## References

[1] Chua M, Wee J, Hui EP, Chan A (2016). Nasopharyngeal carcinoma. Lancet.

[2] Bray F, Ferlay J, Soerjomataram I, Siegel RL, Torre LA, Jemal A (2018). Global cancer statistics 2018: GLOBOCAN estimates of incidence and mortality worldwide for 36 cancers in 185 countries. CA Cancer J Clin.

[3] Elgui de Oliveira D, Müller-Coan BG, Pagano JS (2016). Viral Carcinogenesis Beyond Malignant Transformation: EBV in the Progression of Human Cancers. Trends Microbiol.

[4] Lo EJ, Bell D, Woo JS (2010). Human papillomavirus and WHO type I nasopharyngeal carcinoma. Laryngoscope.

[5] Lee AW, Ma BB, Ng WT, Chan AT (2015). Management of Nasopharyngeal Carcinoma: Current Practice and Future Perspective. J Clin Oncol.

[6] Lin S, Pan J, Han L (2014). Update report of nasopharyngeal carcinoma treated with reduced-volume intensity-modulated radiation therapy and hypothesis of the optimal margin. Radiother Oncol.

[7] Lai SZ, Li WF, Chen L (2011). How does intensity-modulated radiotherapy versus conventional two-dimensional radiotherapy influence the treatment results in nasopharyngeal carcinoma patients. Int J Radiat Oncol Biol Phys.

[8] Zhang MX, Li J, Shen GP (2015). Intensity-modulated radiotherapy prolongs the survival of patients with nasopharyngeal carcinoma compared with conventional two-dimensional radiotherapy: A 10-year experience with a large cohort and long follow-up. Eur J Cancer.

[9] Kam MK, Wong FC, Kwong DL, Sze HC, Lee AW (2014). Current controversies in radiotherapy for nasopharyngeal carcinoma (NPC). Oral Oncol.

[10] Liu SC, Tsang NM, Chiang WC (2013). Leukemia inhibitory factor promotes nasopharyngeal carcinoma progression and radioresistance. J Clin Invest.

[11] Qu C, Zhao Y, Feng G (2017). RPA3 is a potential marker of prognosis and radioresistance for nasopharyngeal carcinoma. J Cell Mol Med.

[12] Chan OS, Sze HC, Lee MC (2017). Reirradiation with intensity-modulated radiotherapy for locally recurrent T3 to T4 nasopharyngeal carcinoma. Head Neck.

[13] Lee SY, Jeong EK, Ju MK (2017). Induction of metastasis, cancer stem cell phenotype, and oncogenic metabolism in cancer cells by ionizing radiation. Mol Cancer.

[14] Kutuk O, Aytan N, Karakas B (2017). Biphasic ROS production, p53 and BIK dictate the mode of cell death in response to DNA damage in colon cancer cells. PLoS One.

[15] Surova O, Zhivotovsky B (2013). Various modes of cell death induced by DNA damage. Oncogene.

[16] Dawood S, Austin L, Cristofanilli M (2014). Cancer stem cells: implications for cancer therapy. Oncology (Williston Park).

[17] Peiris-Pagès M, Martinez-Outschoorn UE, Pestell RG, Sotgia F, Lisanti MP (2016). Cancer stem cell metabolism. Breast Cancer Res.

[18] Vlashi E, Pajonk F (2015). Cancer stem cells, cancer cell plasticity and radiation therapy. Semin Cancer Biol.

[19] Singh SK, Hawkins C, Clarke ID (2004). Identification of human brain tumour initiating cells. Nature.

[20] Abou-Antoun TJ, Hale JS, Lathia JD, Dombrowski SM (2017). Brain Cancer Stem Cells in Adults and Children: Cell Biology and Therapeutic Implications. Neurotherapeutics.

[21] Yang F, Xu J, Tang L, Guan X (2017). Breast cancer stem cell: the roles and therapeutic implications. Cell Mol Life Sci.

[22] Regan JL, Schumacher D, Staudte S (2017). Non-Canonical Hedgehog Signaling Is a Positive Regulator of the WNT Pathway and Is Required for the Survival of Colon Cancer Stem Cells. Cell Rep.

[23] Zhao F, He X, Sun J (2015). Cancer stem cell vaccine expressing ESAT-6-gpi and IL-21 inhibits melanoma growth and metastases. Am J Transl Res.

[24] Wei P, Niu M, Pan S (2014). Cancer stem-like cell: a novel target for nasopharyngeal carcinoma therapy. Stem Cell Res Ther.

[25] Zhang HB, Ren CP, Yang XY (2007). Identification of label-retaining cells in nasopharyngeal epithelia and nasopharyngeal carcinoma tissues. Histochem Cell Biol.

[26] Jiang QP, Yao KT (2010). Isolation and detection of label-retaining cells in a nasopharyngeal carcinoma cell line. Chin J Cancer.

[27] Wang J, Guo LP, Chen LZ, Zeng YX, Lu SH (2007). Identification of cancer stem cell-like side population cells in human nasopharyngeal carcinoma cell line. Cancer Res.

[28] Wang S, Ma N, Zhao W (2016). Inflammation-Related DNA Damage and Cancer Stem Cell Markers in Nasopharyngeal Carcinoma. Mediators Inflamm.

[29] Su J, Xu XH, Huang Q (2011). Identification of cancer stem-like CD44+ cells in human nasopharyngeal carcinoma cell line. Arch Med Res.

[30] Yang CH, Wang HL, Lin YS (2014). Identification of CD24 as a cancer stem cell marker in human nasopharyngeal carcinoma. PLoS One.

[31] Zhuang HW, Mo TT, Hou WJ (2013). Biological characteristics of CD133(+) cells in nasopharyngeal carcinoma. Oncol Rep.

[32] van Schaijik B, Davis PF, Wickremesekera AC, Tan ST, Itinteang T (2018). Subcellular localisation of the stem cell markers OCT4, SOX2, NANOG, KLF4 and c-MYC in cancer: a review. J Clin Pathol.

[33] Luo W, Li S, Peng B, Ye Y, Deng X, Yao K (2013). Embryonic stem cells markers SOX2, OCT4 and Nanog expression and their correlations with epithelial-mesenchymal transition in nasopharyngeal carcinoma. PLoS One.

[34] Krause M, Dubrovska A, Linge A, Baumann M (2017). Cancer stem cells: Radioresistance, prediction of radiotherapy outcome and specific targets for combined treatments. Adv Drug Deliv Rev.

[35] Marie-Egyptienne DT, Lohse I, Hill RP (2013). Cancer stem cells, the epithelial to mesenchymal transition (EMT) and radioresistance: potential role of hypoxia. Cancer Lett.

[36] Skvortsova I, Debbage P, Kumar V, Skvortsov S (2015). Radiation resistance: Cancer stem cells (CSCs) and their enigmatic pro-survival signaling. Semin Cancer Biol.

[37] Chaffer CL, San Juan BP, Lim E, Weinberg RA (2016). EMT, cell plasticity and metastasis. Cancer Metastasis Rev.

[38] Wu SL, Li YJ, Liao K (2017). 2-Methoxyestradiol inhibits the proliferation and migration and reduces the radioresistance of nasopharyngeal carcinoma CNE-2 stem cells via NF-κB/HIF-1 signaling pathway inactivation and EMT reversal. Oncol Rep.

[39] Wang J, Kang M, Wen Q (2017). Berberine sensitizes nasopharyngeal carcinoma cells to radiation through inhibition of Sp1 and EMT. Oncol Rep.

[40] Hu Y, Qi MF, Xu QL (2017). Candidate tumor suppressor ZNF154 suppresses invasion and metastasis in NPC by inhibiting the EMT via Wnt/β-catenin signalling. Oncotarget.

[41] Cai L, Ye Y, Jiang Q (2015). Epstein-Barr virus-encoded microRNA BART1 induces tumour metastasis by regulating PTEN-dependent pathways in nasopharyngeal carcinoma. Nat Commun.

[42] Jun S, Jung YS, Suh HN (2016). LIG4 mediates Wnt signalling-induced radioresistance. Nat Commun.

[43] Wang G, Shen J, Sun J (2017). Cyclophilin A Maintains Glioma-Initiating Cell Stemness by Regulating Wnt/β-Catenin Signaling. Clin Cancer Res.

[44] Zhao Y, Yi J, Tao L (2018). Wnt signaling induces radioresistance through upregulating HMGB1 in esophageal squamous cell carcinoma. Cell Death Dis.

[45] He H, Lin K, Su Y (2018). Overexpression of β-Catenin Decreases the Radiosensitivity of Human Nasopharyngeal Carcinoma CNE-2 Cells. Cell Physiol Biochem.

[46] Polakis P (2012). Wnt signaling in cancer. Cold Spring Harb Perspect Biol.

[47] Li G, Liu Y, Su Z (2013). MicroRNA-324-3p regulates nasopharyngeal carcinoma radioresistance by directly targeting WNT2B. Eur J Cancer.

[48] Li G, Wang Y, Liu Y (2014). miR-185-3p regulates nasopharyngeal carcinoma radioresistance by targeting WNT2B in vitro. Cancer Sci.

[49] Liu C, Li G, Ren S (2017). miR-185-3p regulates the invasion and metastasis of nasopharyngeal carcinoma by targeting WNT2B in vitro. Oncol Lett.

[50] Zhang G, Wang W, Yao C (2017). Radiation-resistant cancer stem-like cell properties are regulated by PTEN through the activity of nuclear β-catenin in nasopharyngeal carcinoma. Oncotarget.

[51] Xia Y, Shen S, Verma IM (2014). NF-κB, an active player in human cancers. Cancer Immunol Res.

[52] Rinkenbaugh AL, Baldwin AS (2016). The NF-κB Pathway and Cancer Stem Cells. Cells.

[53] Li CW, Xia W, Huo L (2012). Epithelial-mesenchymal transition induced by TNF-α requires NF-κB-mediated transcriptional upregulation of Twist1. Cancer Res.

[54] Veuger SJ, Hunter JE, Durkacz BW (2009). Ionizing radiation-induced NF-kappaB activation requires PARP-1 function to confer radioresistance. Oncogene.

[55] Huang T, Yin L, Wu J (2016). MicroRNA-19b-3p regulates nasopharyngeal carcinoma radiosensitivity by targeting TNFAIP3/NF-κB axis. J Exp Clin Cancer Res.

[56] Li LN, Xiao T, Yi HM (2017). MiR-125b Increases Nasopharyngeal Carcinoma Radioresistance by Targeting A20/NF-κB Signaling Pathway. Mol Cancer Ther.

[57] Yuan X, Wu H, Xu H (2015). Notch signaling: an emerging therapeutic target for cancer treatment. Cancer Lett.

[58] Yu S, Zhang R, Liu F, Hu H, Yu S, Wang H (2011). Down-regulation of Notch signaling by a γ-secretase inhibitor enhances the radiosensitivity of nasopharyngeal carcinoma cells. Oncol Rep.

[59] Yu S, Zhang R, Liu F, Wang H, Wu J, Wang Y (2012). Notch inhibition suppresses nasopharyngeal carcinoma by depleting cancer stem-like side population cells. Oncol Rep.

[60] Xie X, Wang H, Jin H (2013). Expression of pAkt affects p53 codon 72 polymorphism-based prediction of response to radiotherapy in nasopharyngeal carcinoma. Radiat Oncol.

[61] Wang MH, Sun R, Zhou XM (2018). Epithelial cell adhesion molecule overexpression regulates epithelial-mesenchymal transition, stemness and metastasis of nasopharyngeal carcinoma cells via the PTEN/AKT/mTOR pathway. Cell Death Dis.

[62] Qu JQ, Yi HM, Ye X (2015). MiRNA-203 Reduces Nasopharyngeal Carcinoma Radioresistance by Targeting IL8/AKT Signaling. Mol Cancer Ther.

[63] Yuan L, Yi HM, Yi H (2016). Reduced RKIP enhances nasopharyngeal carcinoma radioresistance by increasing ERK and AKT activity. Oncotarget.

[64] Ma R, Zhao LN, Yang H (2018). RNA binding motif protein 3 (RBM3) drives radioresistance in nasopharyngeal carcinoma by reducing apoptosis via the PI3K/AKT/Bcl-2 signaling pathway. Am J Transl Res.

[65] Ou J, Pan F, Geng P (2012). Silencing fibronectin extra domain A enhances radiosensitivity in nasopharyngeal carcinomas involving an FAK/Akt/JNK pathway. Int J Radiat Oncol Biol Phys.

[66] Wang D, Wang S, Liu Q, Wang M, Wang C, Yang H (2013). SZ-685C exhibits potent anticancer activity in both radiosensitive and radioresistant NPC cells through the miR-205-PTEN-Akt pathway. Oncol Rep.

[67] Qu C, Liang Z, Huang J (2012). MiR-205 determines the radioresistance of human nasopharyngeal carcinoma by directly targeting PTEN. Cell Cycle.

[68] Port RJ, Pinheiro-Maia S, Hu C (2013). Epstein-Barr virus induction of the Hedgehog signalling pathway imposes a stem cell phenotype on human epithelial cells. J Pathol.

[69] Song Q, Li Y, Zheng X (2013). MTA1 contributes to actin cytoskeleton reorganization and metastasis of nasopharyngeal carcinoma by modulating Rho GTPases and Hedgehog signaling. Int J Biochem Cell Biol.

[70] Chen W, Hu GH (2015). Biomarkers for enhancing the radiosensitivity of nasopharyngeal carcinoma. Cancer Biol Med.

[71] Nicola NA, Babon JJ (2015). Leukemia inhibitory factor (LIF). Cytokine Growth Factor Rev.

[72] Luftig M (2013). Heavy LIFting: tumor promotion and radioresistance in NPC. J Clin Invest.

[73] Lu Y, Liang Y, Zheng X, Deng X, Huang W, Zhang G (2019). EVI1 promotes epithelial-to-mesenchymal transition, cancer stem cell features and chemo-/radioresistance in nasopharyngeal carcinoma. J Exp Clin Cancer Res.

[74] Huang L, Liao L, Wan Y (2016). Downregulation of Annexin A1 is correlated with radioresistance in nasopharyngeal carcinoma. Oncol Lett.

[75] Wei F, Tang L, He Y (2018). BPIFB1 (LPLUNC1) inhibits radioresistance in nasopharyngeal carcinoma by inhibiting VTN expression. Cell Death Dis.

[76] Pan X, Mou J, Liu S (2015). SHP-1 overexpression increases the radioresistance of NPC cells by enhancing DSB repair, increasing S phase arrest and decreasing cell apoptosis. Oncol Rep.

[77] Sun Z, Pan X, Zou Z, Ding Q, Wu G, Peng G (2015). Increased SHP-1 expression results in radioresistance, inhibition of cellular senescence, and cell cycle redistribution in nasopharyngeal carcinoma cells. Radiat Oncol.

[78] Zhou L, Chen HM, Qu S (2018). Reduced QSOX1 enhances radioresistance in nasopharyngeal carcinoma. Oncotarget.

[79] Nie X, Guo E, Wu C (2019). SALL4 induces radioresistance in nasopharyngeal carcinoma via the ATM/Chk2/p53 pathway. Cancer Med.

[80] Zhang B, Qu JQ, Xiao L (2012). Identification of heat shock protein 27 as a radioresistance-related protein in nasopharyngeal carcinoma cells. J Cancer Res Clin Oncol.

[81] Lan R, Huang F, Zhong G (2018). Effects of CKMT1 on radiosensitivity of nasopharyngeal carcinoma cells. Int J Radiat Biol.

[82] Lin H, Chen ZT, Zhu XD (2017). Serum CD166: A novel biomarker for predicting nasopharyngeal carcinoma response to radiotherapy. Oncotarget.

[83] Yang R, Tao ZZ, Huang ML (2018). Knockout of the placenta specific 8 gene radiosensitizes nasopharyngeal carcinoma cells by activating the PI3K/AKT/GSK3β pathway. Am J Transl Res.

[84] Hanahan D, Weinberg RA (2011). Hallmarks of cancer: the next generation. Cell.

[85] Yuan L, Liu ZH, Lin ZR, Xu LH, Zhong Q, Zeng MS (2014). Recurrent FGFR3-TACC3 fusion gene in nasopharyngeal carcinoma. Cancer Biol Ther.

[86] Chung GT, Lung RW, Hui AB (2013). Identification of a recurrent transforming UBR5-ZNF423 fusion gene in EBV-associated nasopharyngeal carcinoma. J Pathol.

[87] Zhong Q, Liu ZH, Lin ZR (2018). The RARS-MAD1L1 Fusion Gene Induces Cancer Stem Cell-like Properties and Therapeutic Resistance in Nasopharyngeal Carcinoma. Clin Cancer Res.

[88] Lee KT, Tan JK, Lam AK, Gan SY (2016). MicroRNAs serving as potential biomarkers and therapeutic targets in nasopharyngeal carcinoma: A critical review. Crit Rev Oncol Hematol.

[89] Atambayeva S, Niyazova R, Ivashchenko A, Pyrkova A, Pinsky I, Akimniyazova A, Labeit S (2017). The Binding Sites of miR-619-5p in the mRNAs of Human and Orthologous Genes. BMC Genomics.

[90] Wu W, Chen X, Yu S, Wang R, Zhao R, Du C (2018). microRNA-222 promotes tumor growth and confers radioresistance in nasopharyngeal carcinoma by targeting PTEN. Mol Med Rep.

[91] Huang D, Bian G, Pan Y (2017). MiR-20a-5p promotes radio-resistance by targeting Rab27B in nasopharyngeal cancer cells. Cancer Cell Int.

[92] Zhao F, Pu Y, Qian L, Zang C, Tao Z, Gao J (2017). MiR-20a-5p promotes radio-resistance by targeting NPAS2 in nasopharyngeal cancer cells. Oncotarget.

[93] Wang T, Dong XM, Zhang FL, Zhang JR (2017). miR-206 enhances nasopharyngeal carcinoma radiosensitivity by targeting IGF1. Kaohsiung J Med Sci.

[94] Zhao L, Tang M, Hu Z (2015). miR-504 mediated down-regulation of nuclear respiratory factor 1 leads to radio-resistance in nasopharyngeal carcinoma. Oncotarget.

[95] Qu JQ, Yi HM, Ye X (2015). MiR-23a sensitizes nasopharyngeal carcinoma to irradiation by targeting IL-8/Stat3 pathway. Oncotarget.

[96] Kang M, Xiao J, Wang J (2016). MiR-24 enhances radiosensitivity in nasopharyngeal carcinoma by targeting SP1. Cancer Med.

[97] Wang S, Pan Y, Zhang R (2016). Hsa-miR-24-3p increases nasopharyngeal carcinoma radiosensitivity by targeting both the 3'UTR and 5'UTR of Jab1/CSN5. Oncogene.

[98] Feng X, Lv W, Wang S, He Q (2018). miR-495 enhances the efficacy of radiotherapy by targeting GRP78 to regulate EMT in nasopharyngeal carcinoma cells. Oncol Rep.

[99] Zhan Y, Zang H, Feng J, Lu J, Chen L, Fan S (2017). Long non-coding RNAs associated with non-small cell lung cancer. Oncotarget.

[100] Wilusz JE, Sunwoo H, Spector DL (2009). Long noncoding RNAs: functional surprises from the RNA world. Genes Dev.

[101] Yang L, Tang Y, He Y (2017). High Expression of LINC01420 indicates an unfavorable prognosis and modulates cell migration and invasion in nasopharyngeal carcinoma. J Cancer.

[102] Sun KY, Peng T, Chen Z, Song P, Zhou XH (2017). Long non-coding RNA LOC100129148 functions as an oncogene in human nasopharyngeal carcinoma by targeting miR-539-5p. Aging (Albany NY).

[103] Su H, Liu L, Zhang Y, Wang J, Zhao Y (2019). Long noncoding RNA NPCCAT1 promotes nasopharyngeal carcinoma progression via upregulating YY1. Biochimie.

[104] Lian Y, Xiong F, Yang L, Bo H, Gong Z, Wang Y, Wei F, Tang Y, Li X, Liao Q, Wang H, Zhou M, Xiang B, Wu X, Li Y, Li X, Chen X, Li G, Guo C, Zeng Z, Xiong W (2018). Long noncoding RNA AFAP1-AS1 acts as a competing endogenous RNA of miR-423-5p to facilitate nasopharyngeal carcinoma metastasis through regulating the Rho/Rac pathway. J Exp Clin Cancer Res.

[105] Guo J, Ma J, Zhao G (2017). Long Noncoding RNA LINC0086 Functions as a Tumor Suppressor in Nasopharyngeal Carcinoma by Targeting miR-214. Oncol Res.

[106] Chak WP, Lung RW, Tong JH (2017). Downregulation of long non-coding RNA MEG3 in nasopharyngeal carcinoma. Mol Carcinog.

[107] Shi B, Wang Y, Yin F (2017). MALAT1/miR-124/Capn4 axis regulates proliferation, invasion and EMT in nasopharyngeal carcinoma cells. Cancer Biol Ther.

[108] Qian W, Ren Z, Lu X (2019). Knockdown of long non-coding RNA TUG1 suppresses nasopharyngeal carcinoma progression by inhibiting epithelial-mesenchymal transition (EMT) via the promotion of miR-384. Biochem Biophys Res Commun.

[109] Cheng Y (2019). FEZF1-AS1 is a key regulator of cell cycle, epithelial-mesenchymal transition and Wnt/β-catenin signaling in nasopharyngeal carcinoma cells. Biosci Rep.

[110] Gao L, Cheng XL, Cao H (2018). LncRNA THOR attenuates cisplatin sensitivity of nasopharyngeal carcinoma cells via enhancing cells stemness. Biochimie.

[111] He Y, Jing Y, Wei F (2018). Long non-coding RNA PVT1 predicts poor prognosis and induces radioresistance by regulating DNA repair and cell apoptosis in nasopharyngeal carcinoma. Cell Death Dis.

[112] Hu X, Jiang H, Jiang X (2017). Downregulation of lncRNA ANRIL inhibits proliferation, induces apoptosis, and enhances radiosensitivity in nasopharyngeal carcinoma cells through regulating miR-125a. Cancer Biol Ther.

[113] Ye M, Huang T, Ni C, Yang P, Chen S (2017). Diagnostic Capacity of RASSF1A Promoter Methylation as a Biomarker in Tissue, Brushing, and Blood Samples of Nasopharyngeal Carcinoma. EBioMedicine.

[114] Li L, Zhang Y, Fan Y (2015). Characterization of the nasopharyngeal carcinoma methylome identifies aberrant disruption of key signaling pathways and methylated tumor suppressor genes. Epigenomics.

[115] Wang S, Zhang R, Claret FX, Yang H (2014). Involvement of microRNA-24 and DNA methylation in resistance of nasopharyngeal carcinoma to ionizing radiation. Mol Cancer Ther.

[116] Li HP, Chang YS (2003). Epstein-Barr virus latent membrane protein 1: structure and functions. J Biomed Sci.

[117] Chen YR, Liu MT, Chang YT, Wu CC, Hu CY, Chen JY (2008). Epstein-Barr virus latent membrane protein 1 represses DNA repair through the PI3K/Akt/FOXO3a pathway in human epithelial cells. J Virol.

[118] Lu J, Tang M, Li H (2016). EBV-LMP1 suppresses the DNA damage response through DNA-PK/AMPK signaling to promote radioresistance in nasopharyngeal carcinoma. Cancer Lett.

[119] Yang CF, Peng LX, Huang TJ (2014). Cancer stem-like cell characteristics induced by EB virus-encoded LMP1 contribute to radioresistance in nasopharyngeal carcinoma by suppressing the p53-mediated apoptosis pathway. Cancer Lett.

[120] Yang F, Liu Q, Hu CM (2015). Epstein-Barr virus-encoded LMP1 increases miR-155 expression, which promotes radioresistance of nasopharyngeal carcinoma via suppressing UBQLN1. Eur Rev Med Pharmacol Sci.

[121] Wu Q, Han T, Sheng X, Zhang N, Wang P (2018). Downregulation of EB virus miR-BART4 inhibits proliferation and aggressiveness while promoting radiosensitivity of nasopharyngeal carcinoma. Biomed Pharmacother.

[122] Wu T, Dai Y (2017). Tumor microenvironment and therapeutic response. Cancer Lett.

[123] Chen J, Yang P, Xiao Y (2017). Overexpression of α-sma-positive fibroblasts (CAFs) in Nasopharyngeal Carcinoma Predicts Poor Prognosis. J Cancer.

[124] Yu Y, Ke L, Lv X (2018). The prognostic significance of carcinoma-associated fibroblasts and tumor-associated macrophages in nasopharyngeal carcinoma. Cancer Manag Res.

[125] Zhang H, Yue J, Jiang Z (2017). CAF-secreted CXCL1 conferred radioresistance by regulating DNA damage response in a ROS-dependent manner in esophageal squamous cell carcinoma. Cell Death Dis.

[126] Zhang H, Hua Y, Jiang Z (2019). Cancer-associated Fibroblast-promoted LncRNA DNM3OS Confers Radioresistance by Regulating DNA Damage Response in Esophageal Squamous Cell Carcinoma. Clin Cancer Res.

[127] Wang S, Ma N, Kawanishi S (2014). Relationships of alpha-SMA-positive fibroblasts and SDF-1-positive tumor cells with neoangiogenesis in nasopharyngeal carcinoma. Biomed Res Int.

[128] Harada H, Kizaka-Kondoh S, Li G (2007). Significance of HIF-1-active cells in angiogenesis and radioresistance. Oncogene.

[129] Meijer TW, Kaanders JH, Span PN, Bussink J (2012). Targeting hypoxia, HIF-1, and tumor glucose metabolism to improve radiotherapy efficacy. Clin Cancer Res.

[130] Hsu HW, Wall NR, Hsueh CT (2014). Combination antiangiogenic therapy and radiation in head and neck cancers. Oral Oncol.

[131] Tang L, Wei F, Wu Y, He Y, Shi L, Xiong F, Gong Z, Guo C, Li X, Deng H, Cao K, Zhou M, Xiang B, Li X, Li Y, Li G, Xiong W, Zeng Z (2018). Role of metabolism in cancer cell radioresistance and radiosensitization methods. J Exp Clin Cancer Res.

[132] Eckert F, Zwirner K, Boeke S, Thorwarth D, Zips D, Huber SM (2019). Rationale for Combining Radiotherapy and Immune Checkpoint Inhibition for Patients With Hypoxic Tumors. Front Immunol.

[133] Ooft ML, van Ipenburg JA, Braunius WW, Zuur CI, Koljenović S, Willems SM (2017). Prognostic role of tumor infiltrating lymphocytes in EBV positive and EBV negative nasopharyngeal carcinoma. Oral Oncol.

[134] Zhang L, MacIsaac KD, Zhou T (2017). Genomic Analysis of Nasopharyngeal Carcinoma Reveals TME-Based Subtypes. Mol Cancer Res.

[135] Huang S, Tsao SW, Tsang CM (2018). Interplay of Viral Infection, Host Cell Factors and Tumor Microenvironment in the Pathogenesis of Nasopharyngeal Carcinoma. Cancers (Basel).

[136] Kedersha N, Ivanov P, Anderson P (2013). Stress granules and cell signaling: more than just a passing phase. Trends Biochem Sci.

[137] Anderson P, Kedersha N, Ivanov P (2015). Stress granules, P-bodies and cancer. Biochim Biophys Acta.

[138] Thoms J, Bristow RG (2010). DNA repair targeting and radiotherapy: a focus on the therapeutic ratio. Semin Radiat Oncol.

[139] El-Naggar AM, Sorensen PH (2018). Translational control of aberrant stress responses as a hallmark of cancer. J Pathol.

[140] Moeller BJ, Cao Y, Li CY, Dewhirst MW (2004). Radiation activates HIF-1 to regulate vascular radiosensitivity in tumors: role of reoxygenation, free radicals, and stress granules. Cancer Cell.

[141] Fournier MJ, Coudert L, Mellaoui S (2013). Inactivation of the mTORC1-eukaryotic translation initiation factor 4E pathway alters stress granule formation. Mol Cell Biol.

[142] Wang W, Wen Q, Luo J (2017). Suppression Of β-catenin Nuclear Translocation By CGP57380 Decelerates Poor Progression And Potentiates Radiation-Induced Apoptosis in Nasopharyngeal Carcinoma. Theranostics.

[143] Protter D, Parker R (2016). Principles and Properties of Stress Granules. Trends Cell Biol.

